# Impacts of Crystal Quality on Carrier Recombination and Spin Dynamics in (110)-Oriented GaAs/AlGaAs Multiple Quantum Wells at Room Temperature

**DOI:** 10.3390/mi12091112

**Published:** 2021-09-16

**Authors:** Satoshi Iba, Ryogo Okamoto, Koki Obu, Yuma Obata, Yuzo Ohno

**Affiliations:** 1 Research Center for Emerging Computing Technologies, National Institute of Advanced Industrial Science and Technology (AIST), Tsukuba 305-8568, Japan; ono.yuzo.gb@u.tsukuba.ac.jp; 2 Graduate School of Pure and Applied Sciences, University of Tsukuba, Tsukuba 305-8573, Japan; ogoyrokamoto@gmail.com (R.O.); kusataipu38@yahoo.co.jp (K.O.); imperial.vortex7@gmail.com (Y.O.)

**Keywords:** molecular beam epitaxy, GaAs (110), photoluminescence, spin relaxation

## Abstract

We have systematically investigated the structural properties, carrier lifetimes, namely, photoluminescence (PL) lifetimes (*τ*_PL_), and electron spin relaxation times (*τ*_s_) in (110) GaAs/AlGaAs multiple quantum wells (MQWs) by using time-resolved PL measurements. The MQWs were grown by molecular beam epitaxy within a wide range of the growth temperature *T*_g_ (430–600 °C) and a high V/III flux ratio using As_2_. At 530 °C < *T*_g_ < 580 °C, we found that the quality of the heterointerfaces is significantly improved, resulting in *τ*_PL_~40 ns at RT, one order of magnitude longer than those reported so far. Long *τ*_s_ (~6 ns) is also observed at RT.

## 1. Introduction

Semiconductor spintronics has attracted growing interest in the last three decades [1]. In nonmagnetic semiconductors, the retention time of electron spin polarization, i.e., the spin relaxation time (*τ*_s_), is one of the most important indexes because it significantly affects the performance of spintronic devices such as spin transistors [2] and spin-photonics devices [3,4,5]. For III-V compound semiconductors, *τ*_s_ in bulk and (100)-oriented quantum wells (QWs) have been extensively investigated so far [6,7]. In the last two decades, on the other hand, much attention has been focused on spin dynamics in non-(100)-oriented QWs, where electron spins have apparently different behaviors from those in (100) QWs [8]. As such, (110) QWs are considered to be one of several promising platforms with applications for spin-photonic devices, as well as studies of spin physics [9,10,11,12,13], due to long *τ*_s_ of about several nanoseconds at room temperature (RT) [14]. The key parameter to induce such a long *τ*_s_ is the Dresselhaus-type effective magnetic fields (*B*_eff_) generated by spin–orbit interaction via the inversion asymmetry of crystal structure in zincblende III-V compound semiconductors. The direction and the magnitude of *B*_eff_ depend on the electron wavevector, and this allows us to control the arrangement of *B*_eff_ by changing the direction of quantum confinement of electron wavefunctions. In (100) QWs, which are widely used in conventional electronic and photonic devices, *B*_eff_ are in in-plane directions for all the in-plane wavevectors. This results in the random precession of electron spins initially aligned to the out-of-plane directions, and the fast relaxation of the ensemble of electron spins. This process is called the D’yakonov–Perel (DP) spin relaxation mechanism [7]. In contrast with (100) QWs, the distribution of *B*_eff_ in wavevector space for (110) QWs is uniaxial anisotropy oriented in out-of-plane direction. This leads to the suppression of the spin precession for electron spins aligned to out-of-plane direction [15]. Therefore, the out-of-plane *B*_eff_ induced by the Dresselhaus spin–orbit coupling provides *τ*_s_ in the order of nanoseconds at RT, which is an order of magnitude longer than that of (100) QWs [14]. Taking advantage of these excellent features, slow light propagation via coherent population oscillation [16], long-distance spin transport [17], *τ*_s_ modulation by introducing superlattice structures [18] as well as by applying gate vias [19], and circularly polarized lasing in vertical cavity surface emitting laser by optical spin injection [20] have been demonstrated in GaAs-based (110) QWs at RT.

To develop spin-photonic devices, we should also pay attention to the lifetime of carriers at RT, i.e., photoluminescence (PL) lifetime (*τ*_PL_), which is one of the indicators of the crystal quality. There are, however, only a few studies reporting *τ*_PL_ in undoped (110) GaAs/AlGaAs QWs, the value of which is only several nanoseconds [21,22,23]. This value is an order of magnitude shorter than those observed in high-quality (100) GaAs/AlGaAs QWs [24,25]. This indicates that there is room for improvement in crystal growth technology for (110) GaAs/AlGaAs QWs. To develop high-quality materials is indispensable for device applications. In crystal growth on GaAs (110) substrates, it is challenging to keep stoichiometry composition and incorporate constituent atoms in the lattice simultaneously, due to the non-polar surface of the GaAs (110) plane [26]. The deviation from stoichiometry and inadequate incorporation of adatoms, which are significantly affected by the growth temperature (*T*_g_) in molecular beam epitaxy (MBE), can cause various crystal defects such as point defects and stacking faults. These degrade the optical properties of the epitaxial layers. To overcome the difficulties, we prepared (110) GaAs/AlGaAs QWs by MBE with varying *T*_g_ in a wide range under the irradiation of large numbers of As atoms. Systematic investigations of the structural and optical properties revealed that *τ*_PL_ exhibits the longest value of 40 ns at *T*_g_ = 580 °C, which is one order of magnitude longer than those with conventional growth conditions for GaAs (110). The longest *τ*_s_ is also obtained at 530 °C < *T*_g_ < 580 °C.

## 2. Experimental Methods

All the samples studied here were grown on epi-ready, semi-insulating (110) on-axis GaAs substrates by an Eiko EV-100 MBE system. Seven samples consisted of 10 nm GaAs cap layers, 60 periods of 10 nm GaAs/10 nm Al_0.3_Ga_0.7_As multiple quantum wells (MQWs), and 300 nm Al_0.3_Ga_0.7_As/100 nm GaAs buffer layers. The MQW structure was adopted to obtain sufficient PL intensity and to facilitate the time-resolved PL measurements. Prior to the growth, a native oxide on the surface was removed by heating the substrate up to 610 °C under As_2_ exposure. The growth conditions used for all layers are as follows: growth rate of GaAs was 0.5 µm/h, *T*_g_ was varied from 432 to 600 °C, and beam equivalent pressure (BEP) ratio of As_2_ to Ga was set at 80, measured by using a nude ion gauge. The As_2_ beam was generated by using a valved cracking cell. Careful consideration of growth conditions is important for high-quality growth on GaAs (110). Epitaxial growth on (110) surfaces is known to be more difficult than that on (100) surfaces [27]. Widely used growth conditions for growth on (100) surfaces (*T*_g_~580 °C and BEP ratio of As_4_ to Ga~10) lead to poor (110) surface morphology. It has been pointed out that the optimum growth of smooth GaAs (110) layers requires a relatively high As_4_/Ga BEP ratio (40–80) and low growth temperatures (<500 °C). However, such low temperatures result in poor PL properties. Therefore, in this study, we attempted film growth with a wide range of *T*_g_ (432–600 °C) under a high BEP ratio. Here, we mention the importance of using As_2_ as the arsenic species. Since both Ga and As atoms coexist on non-polar GaAs (110) surfaces, the arsenic incorporation coefficient during the growth of GaAs decreases to 50% or less of that on (100) surface when As_4_ is used [26]. With the use of As_2_, on the other hand, the incorporation coefficient is improved about twice as much as in the case of As_4_ [26]. It should be noted that the detection sensitivity of the nude ion gauge is different between As_2_ and As_4_. In our experiments, the cracking temperature was raised from 600 to 900 °C to change the main arsenic species from As_4_ to As_2_. Then, while the temperature of the As source was fixed, the As beam flux detected by the nude ion gauge decreased to one-fifth. Therefore, the number of As atoms for the BEP ratio of As_2_/Ga = 80 corresponded to five times as much as As_4_/Ga = 80. It will be shown in the next section that the irradiation of such a large number of As atoms is important for preventing As deficiency on the growth surface to conduct stoichiometric film growth.

For structural analysis, we carried out reflection high-energy electron diffraction (RHEED), atomic force microscopy (AFM), X-ray diffraction (XRD), and transmission electron microscope (TEM) measurements. To evaluate static optical and structural properties, continuous wave (CW) PL measurements were conducted. Carrier and spin dynamics were characterized by time-resolved PL (TRPL) measurements. In the CW-PL measurements, samples were excited by a diode-pumped solid-state (DPSS) laser. The laser wavelength was 532 nm (2.331 eV), which can excite not only GaAs well layers but also Al_0.3_Ga_0.7_As barrier layers whose band gap energy is 1.802 eV at 300 K. The spot size was about 100 µm diameter, and the intensity was 0.5 mW. In the TRPL measurements, samples were excited by right circularly polarized pump pulses generated by a picosecond wavelength-tunable, mode-locked Ti:Al_2_O_3_ laser. The diameter of the laser spot was about 60 µm. The laser’s average intensity was 1 mW. The excitation wavelength was set at about 70 nm shorter than that of the PL peak, which means that electron–hole pairs are excited only within the QW region.

**Figure 1:**
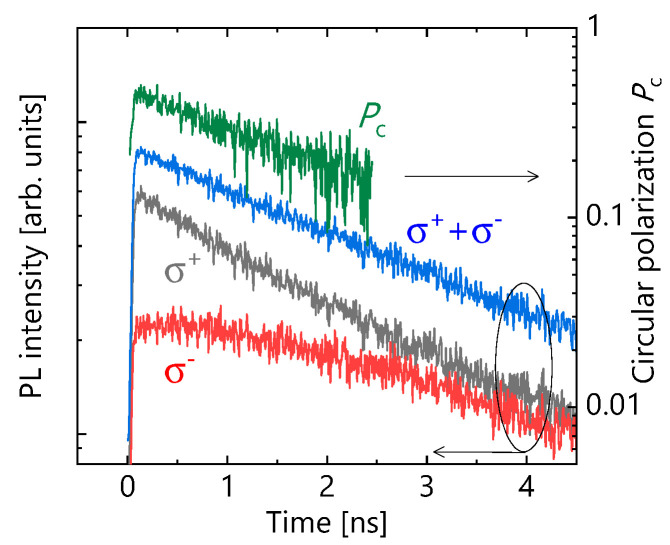
Typical results of polarization- and time-resolved photoluminescence (PL) decay signal for the sample grown at growth temperatures (*T*_g_) of 432 °C measured at room temperature (RT). Circular polarization (*P*_c_) is given by $\left(I_{\sigma +}-I_{\sigma -}\right)/\left(I_{\sigma +}+I_{\sigma -}\right)$ , where $I_{\sigma +}$ and $I_{\sigma -}$ represent right- and left-circularly polarized PL components under *σ*+ excitation, respectively.

To evaluate *τ*_PL_ and *τ*_s_, the TRPL measurements were carried out. In the measurements, spin-polarized electrons—which were excited to the state of the conduction band by right circularly polarized (*σ*+) excitation pulses through the spin-dependent optical selection rules—radiatively decayed to the state of the valence band. The resulting PL was partially circularly polarized, reflecting the degree of spin polarization of the recombination electrons. Right-($I_{\sigma +}\left(t\right)$) and left-($I_{\sigma -}\left(t\right)$) circularly polarized components of the PL were separately converted to orthogonal linearly polarized light by using a quarter-wave plate and a linear polarizer. The PLs were finally measured by using a streak camera through a 25 cm monochromator. As a typical result, the TRPL data for the sample with *T*_g_ = 432 °C are shown in Figure 1. *τ*_PL_ is given by fitting $I_{\sigma +}\left(t\right)+I_{\sigma -}\left(t\right)$ with a single exponential decay function~$\;\exp\left(-t/\tau_{PL}\right)$. From the calculated temporal circular polarization (*P*_c_) of the PLs, $P_{c}\left(t\right)=\left(I_{\sigma +}-I_{\sigma -}\right)/\left(I_{\sigma +}+I_{\sigma -}\right)$, we obtained *τ*_s_ by using a fitting function defined as $P_{c}\left(t\right)\;\sim \;\exp\left(-2t/\tau_{s}\right)$ [28]. Here, we should note that the hole spins are practically unpolarized, due to their short spin relaxation times [29]. The circular polarization observed for the PL therefore comes from the spin polarization of electrons in the conduction band.

## 3. Results and Discussions

### 3.1. Characterizations of Sample Structures and Static Photoluminescence

**Figure 2:**
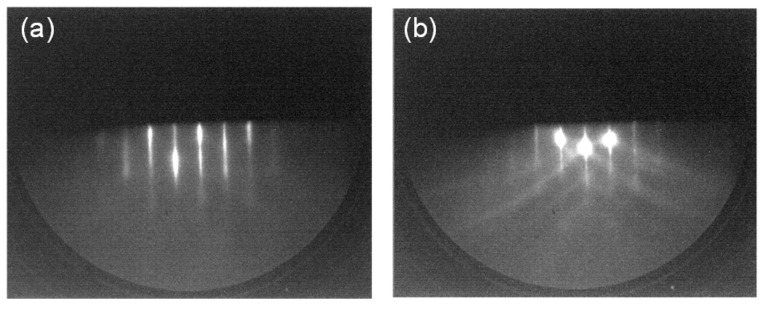
$\left[1\bar{1}0\right]$ azimuth RHEED patterns from (**a**) a GaAs (110) substrate after desorption of a native oxide, (**b**) a GaAs (110) cap layer on the MQW structure with *T*_g_ = 580 °C. Both patterns indicate (1 × 1) non-reconstructed surface.

This section describes sample structure analysis and static PL properties by RHEED, AFM, XRD, CW-PL, and TEM. First, we will explain surface analysis provided by the RHEED measurements. Since the RHEED patterns did not show growth temperature dependence, as a typical result, RHEED images before and after the growth of a sample with *T*_g_ = 580 °C are shown in Figure 2a,b. The characteristic (1 × 1) patterns reflecting bulk periodicity were observed before and after growth, indicating that surface reconstruction did not occur, which is the same as the previous report [26]. Thus, the lesser amount of information obtained from the RHEED measurements on the GaAs (110) surface compared to the widely used GaAs (100) surface makes crystal growth on the GaAs (110) surface difficult.

**Figure 3:**
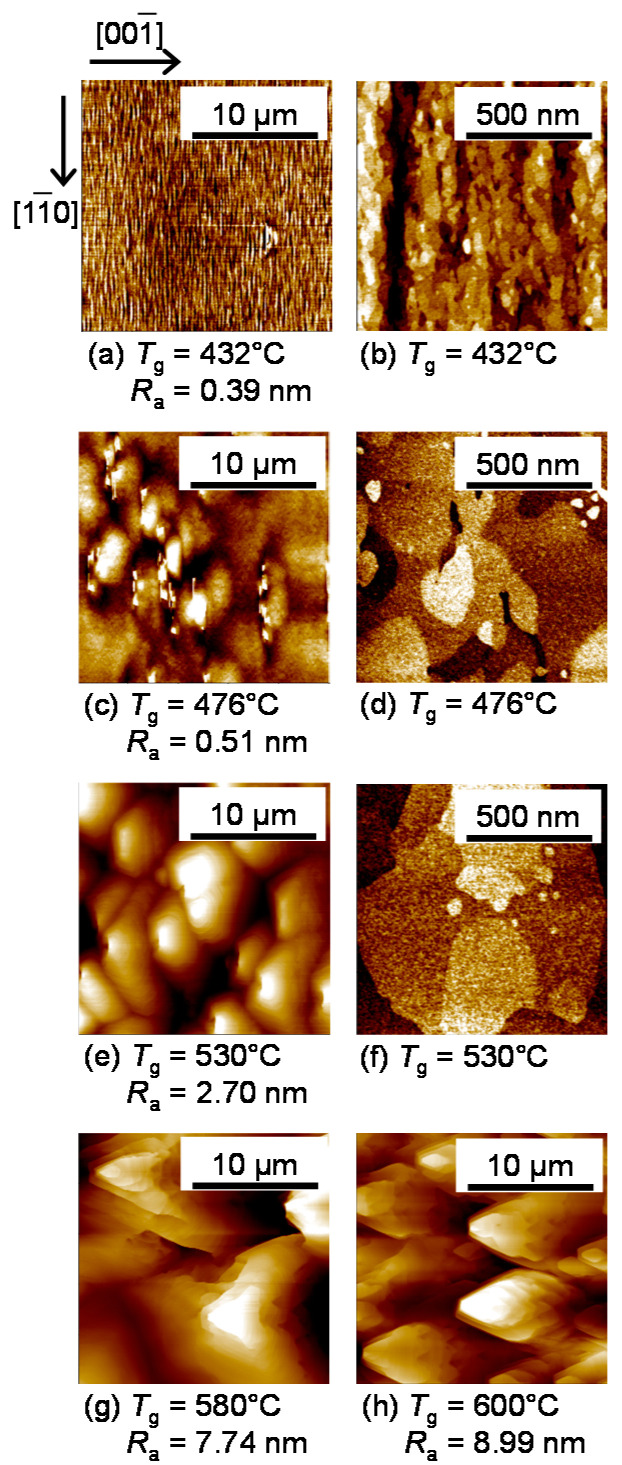
Atomic force microscopy (AFM) images with a scan size of 20 × 20 µm^2^ in (**a**,**c**,**e**,**g**,**h**), and of 1 × 1 µm^2^ in (**b**,**d**,**f**), for the surface of the samples grown at given *T*_g_. The value of the average surface roughness (*R*_a_) of each sample is also shown in the figures. Crystal orientation is indicated by the arrows in (**a**).

The surface morphology of the GaAs cap layer of the sample measured by AFM will be presented. Figure 3a,c,e,g,h are wide-area AFM images (20 × 20 µm^2^) for the samples with *T*_g_ = 432, 476, 530, 580, and 600 °C, respectively. In the sample with *T*_g_ = 432 °C, as shown in Figure 3a, a vertically long pattern (along the $\left[1\bar{1}0\right]$) direction) with a height of about 1 nm was observed. When *T*_g_ was raised to 530 °C, the shape changed to a rounded pattern, and the height of the pattern increased to 5 nm. When *T*_g_ was raised further to 580 °C, the shape changed to a triangle, and its height increased significantly to 40 nm. Tok et al. reported that such triangular patterns can be induced by the local agglomeration and clustering of Ga atoms due to As deficiency [27]. In the sample grown at 600 °C, the density of the triangular pattern with a height of about 40 nm increased. The value of the average surface roughness (*R*_a_) of each sample is shown in the figures. *R*_a_ increased with *T*_g_, reaching about 9 nm when *T*_g_ was 600 °C. Nevertheless, the surfaces were mirror-like, not clouded, which is desirable for the PL measurements. We note here that, in the previous report where As_4_ was used with BEP ratio of about 80, the triangular patterns appeared at *T*_g_ = 480 °C [21], whereas not observed when *T*_g_ < 580 °C in the present study. This means that using As_2_ and a high As_2_/Ga BEP ratio successfully suppressed As deficiency and Ga agglomeration even at higher growth temperatures (500 °C < *T*_g_ < 580 °C) resulting in improvements in surface morphology. Above 580 °C, on the other hand, a significant decrease in the As incorporation coefficient [26] caused As deficiency on the growth surface, forming triangular patterns. The AFM images with a narrow area (1 × 1 µm^2^) of the samples with *T*_g_ = 432, 476, and 530 °C are shown in Figure 3b,d,f. Step-and-terrace structures having a vertically long pattern with a height of 1–2 monolayers were observed in the sample with *T*_g_ = 432 °C. As *T*_g_ increased, the terrace shape became isotropic, and the size increased significantly from several tens of nm to about 1µm. This suggests that for low *T*_g_ < 476 °C, the anisotropic surface diffusion of Ga atoms was induced, which obeys the difference of the migration barrier of Ga atoms between [1] and $\left[1\bar{1}0\right]$ in-plane direction [30]. On the other hand, the diffusion became isotropic due to the high thermal energy as *T*_g_ increased.

**Figure 4:**
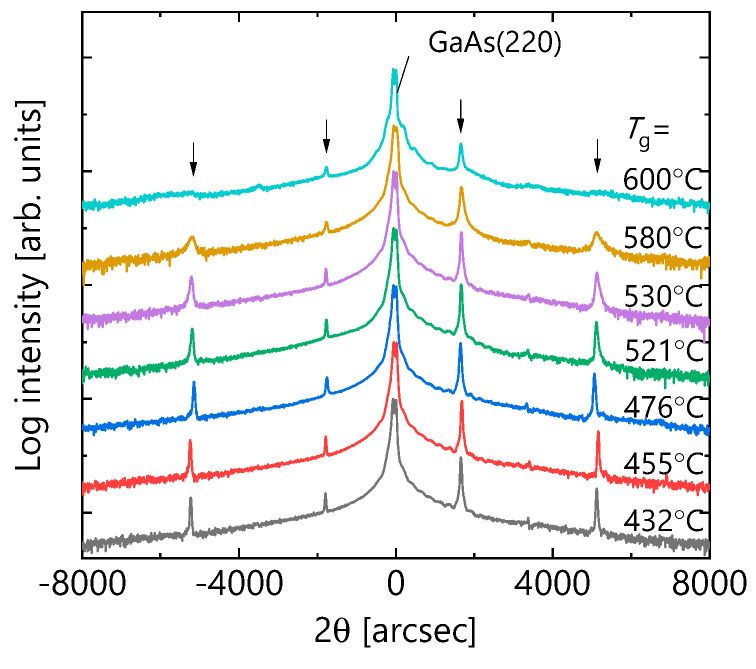
X-ray diffraction (XRD) profiles of θ-2θ scan at the (220) diffraction for the samples with given *T*_g_. The x-axis is the angle relative to the diffraction angle of GaAs. The satellite peaks derived from the periodic structure of multiple quantum wells (MQWs) are indicated by the arrows.

Next, the results of XRD measurements are discussed. The data of θ-2θ scan at the (220) diffraction of each sample is shown are Figure 4. In all the samples, satellite peaks reflecting the periodicity of the MQW structure was detected at the same angle. This reveals that the average thickness of the GaAs wells and AlGaAs barriers are the same for all samples. However, it can be seen that the linewidths of the satellite peaks were remarkably widened, and the peak intensities were lowered in the samples with *T*_g_ = 580 and 600 °C. This indicates that the fluctuation of the thicknesses between layers is remarkably large in these samples.

**Figure 5:**
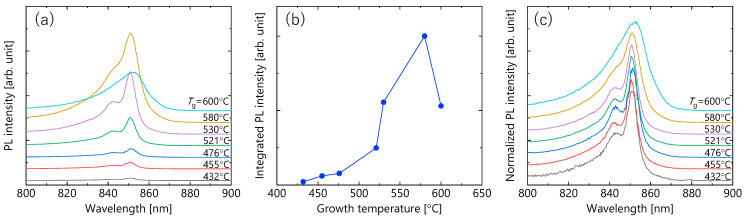
The data of continuous wave (CW)-PL measurements performed at RT. (**a**) PL spectra for the samples with given *T*_g_, (**b**) integrated PL intensity plotted as a function of *T*_g_, and (**c**) PL spectra normalized by the peak intensity for each spectrum depicted in (**a**).

We next explain the results of the CW-PL measurements at RT. Figure 5a shows *T*_g_ dependence in the PL spectra. For all the samples, the PL peaks appeared at the same wavelength. Since the PL wavelength reflects the quantum confinement of carriers, Figure 5a indicates that the quantum structures with the same average well width are formed with different *T*_g_. This seems to be consistent with the XRD analysis showing satellite peaks at the same angle, as shown in Figure 4. The integrated PL intensity of each sample is summarized in Figure 5b. The integrated PL intensity had a peak behavior. When *T*_g_ was raised from 432 to 580 °C, the PL intensity increased about 50 times, and then for *T*_g_ = 600 °C it decreased to about a half of that of 580 °C. The PL intensity is determined by the competition between radiative and non-radiative recombination processes [31]. Therefore, the significant increase in the PL intensity suggests that the non-radiative recombination process is remarkably suppressed by increasing *T*_g_. This interpretation will be explained in detail in the next section. Figure 5c shows PL spectra normalized by peak intensity in each spectrum. There was almost no change in the spectral shape in *T*_g_ range of 432 to 530 °C. The main peak at 851 nm corresponds to the electron-heavy hole transition, and the 843 nm sub-peak corresponds to the electron–light hole transition of their ground state. These measured PL wavelengths almost match the transition wavelengths calculated from given QW structures. The spectral linewidth began to widen at *T*_g_ = 580 °C. This is attributed to the variation in the transition energy, reflecting thickness fluctuations of GaAs well layers in the in-plane and stacking direction in the photoexcited region, which is similar to the *T*_g_ dependence observed by the XRD and AFM measurements.

**Figure 6:**
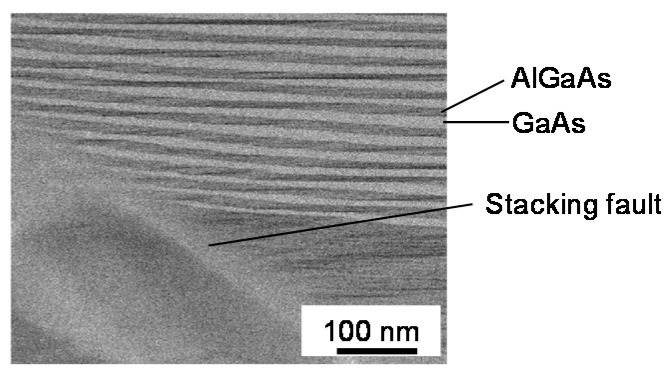
Cross-sectional transmission electron microscope (TEM) image for the sample grown at *T*_g_ = 600 °C. The scan area is beneath the apex of the triangular pattern observed in Figure 3h. In the striped pattern, dark (light) gray areas represent AlGaAs (GaAs) layers. The observed stacking fault is along the <111> direction.

To visually verify the structure fluctuations at higher *T*_g_, cross-sectional TEM analysis was carried out for the sample grown at 600 °C. The scan area depicted in Figure 6 is beneath the apex of the triangular pattern observed in Figure 3h. Stacking faults, which can be caused by the deviations from stoichiometry [32], were formed diagonally, and the thicknesses of GaAs well and AlGaAs barrier layers fluctuated by several nm or more. Such film thickness fluctuations caused a decline in satellite peaks in XRD data in Figure 4, and an increase in the PL linewidth in Figure 5c. Since the stacking faults and point defects that exist around them can act as non-radiative recombination centers, it is reasonable that the PL intensity decreased in a 600 °C sample with many triangular patterns having stacking faults.

### 3.2. Carrier and Spin Dynamics

**Figure 7:**
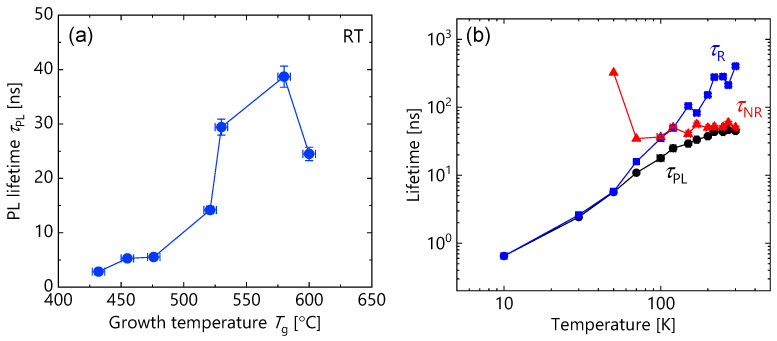
(**a**) Growth temperature dependence of PL lifetimes (*τ*_PL_) measured at RT. (**b**) Temperature dependence of PL lifetimes (*τ*_PL_; solid circles), radiative recombination time (*τ*_R_; solid squares) and non-radiative recombination time (*τ*_NR_; solid triangles) for the sample grown at *T*_g_ = 580 °C.

In this section, we investigate the carrier and spin dynamics measured by the TRPL method. Figure 7a shows the *T*_g_ dependence of *τ*_PL_ measured at RT. Similar to *T*_g_ dependence of CW-PL intensity (shown in Figure 5b), *τ*_PL_ increased with *T*_g_ monotonously up to *T*_g_ = 580 °C, and then decreased with further increases in *T*_g_. The maximum *τ*_PL_, 40 ns, at *T*_g_ = 580 °C is the longest value that has been reported in (110) GaAs/AlGaAs MQWs so far, and comparable to that for high-quality (100) MQWs [24,25]. To investigate carrier dynamics in detail, we extracted the radiative recombination time (*τ*_R_) and the non-radiative recombination time (*τ*_NR_) from the temperature dependence of *τ*_PL_ and PL intensity (*I*_PL_). *τ*_PL_ and *I*_PL_ are given by the following equations using *τ*_R_ and *τ*_NR_, respectively [31].

(1) $$\frac{1}{\tau_{PL}}=\frac{1}{\tau_{R}}+\frac{1}{\tau_{NR}}$$

(2) IPL=1/τR1/τR+1/τ
NR

Figure 7b shows the temperature dependence of *τ*_R_ and *τ*_NR_ obtained from Equations (1) and (2), together with *τ*_PL_ and *I*_PL_ measured at each temperature for the sample with *T*_g_ = 580 °C. It was found that *τ*_NR_ became shorter than *τ*_R_ above 150 K, resulting in significantly dominating *τ*_PL_, namely, *τ*_PL_~*τ*_NR_. Similar behavior was observed for all the samples. Accordingly, the increase in *τ*_NR_ (~*τ*_PL_) at RT for a range of *T*_g_ from 432 to 580 °C in Figure 7a means the decrease in the density of non-radiative recombination center. In (100) GaAs/AlGaAs MQWs, it has been pointed out that the interface non-radiative recombination caused by lattice defects and incorporated impurities near the GaAs/AlGaAs interface determines the *τ*_NR_ at RT [24,25]. Therefore, we should consider that the increase in *τ*_NR_ is attributed to the suppression of interface non-radiative recombination, namely, improvements in interface quality by increasing *T*_g_ to 580 °C. The increasing tendency of terrace size in Figure 3b,d,f may support this interpretation. On the other hand, the drop in *τ*_NR_ (*τ*_PL_) at *T*_g_ = 600 °C presumably originated from the non-radiative recombination centers induced by a large number of stacking faults underneath triangular patterns (see Figure 3h and Figure 6) and point defects around the stacking faults, rather than the effect of improving the interface quality. Therefore, MBE growth of optically high-quality (110) GaAs/AlGaAs MQWs is possible by setting moderately higher *T*_g_ (>500 °C), although it has been believed that low *T*_g_ (<500 °C) is preferable for crystal growth on GaAs (110) [33].

**Figure 8:**
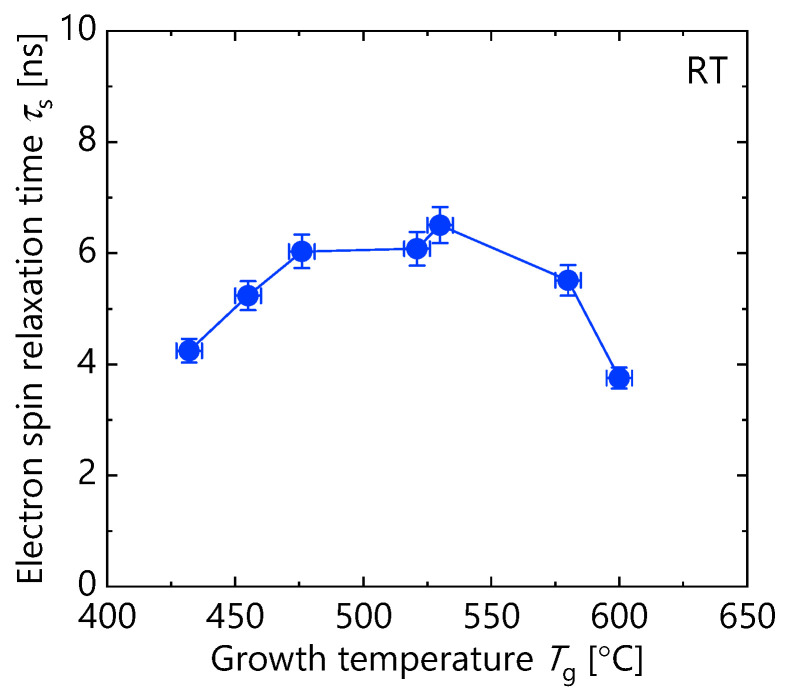
Growth temperature dependence of electron spin relaxation times (*τ*_s_) measured at RT.

Finally, the *T*_g_ dependence of *τ*_s_ obtained at RT by circularly polarized TRPL measurements is discussed. Figure 8 shows *τ*_s_ as a function of *T*_g_. *τ*_s_ increased with *T*_g_ up to 476 °C, then saturated to 530 °C. The longest *τ*_s_ (>6 ns) was obtained around *T*_g_ = 530 °C. On the other hand, the decrease in *τ*_s_ was observed when *T*_g_ ≥ 580 °C. As explained in the previous section, the fluctuation of the MQW structure was remarkable for samples with *T*_g_ = 580 and 600 °C. As can be seen from the cross-sectional TEM image of Figure 6, there were some regions where the interfaces of MQW faced non-(110) plane direction. The quantum confinement with tilting from the (110) orientation caused an in-plane component of *B*_eff_ induced by the Dresselhaus spin–orbit coupling. This promoted the relaxation of spins initially aligned along the out-of-plane direction [22]. This is surely the mechanism by which the *τ*_s_ decreased in high-*T*_g_ samples.

Here, we discuss the mechanism of increasing *τ*_s_ for *T*_g_ ≤ 476 °C. To date, intersubband scattering mechanism [9], and Bir–Aronov–Pikus (BAP) mechanism [14,34] have been proposed for electron spin relaxation mechanisms in (110) GaAs/AlGaAs MQWs at RT. The former depends on the electron concentration and the intersubband energy gap, and the latter depends on the hole concentration. The macroscopic structures of the samples prepared here were almost the same, as shown in the XRD and CW-PL data. In addition, the excitation intensity in the TRPL measurements was the same for all samples, resulting in the same concentrations of the photoexcited electrons and holes for each our sample. Therefore, the possibility that these spin relaxation mechanisms modulate *τ*_s_ should be negligible.

One can come up with following possible scenario: interface imperfections in the (110) MQWs cause additional spin relaxation. There are two candidates for interface imperfections: an interface compositional gradient layer, and interface crystal defects including impurities. Regarding the former, Karimov et al. reported a calculation result that *τ*_s_ is reduced to 1/30 by introducing an interface compositional gradient layer with the thickness of two monolayers [35]. In contrast, it is reported that the Rashba split energy that induces in-plane *B*_eff_ is negligibly small for the MQW having compositional variations, which means that *τ*_s_ is not modulated [36,37]. Since whether the compositional gradient layers are present or not is not sure in our samples, we do not mention their effects on *τ*_s_.

Regarding the latter, the lattice defects and impurities near interfaces may induce random potential, which causes enhancement of spin relaxation by the random Rashba effect [38,39]. As discussed above, the *T*_g_ dependence of *τ*_PL_ at RT, which corresponds to *τ*_NR_, suggests that the number of the lattice defects and impurities near the interfaces decrease when *T*_g_ increases. Therefore, it is natural that *τ*_s_ has *T*_g_ dependence. Figure 8 shows the weak *T*_g_ dependence of *τ*_s_, compared to that of *τ*_PL_. This difference originated from the fact that *τ*_PL_ directly reflects the lattice defects and impurities near interfaces, whereas *τ*_s_ is indirectly related to the interface quality. The DP mechanism induced by the Rashba spin–orbit coupling through the compositional gradient layers, and/or spin relaxation mechanisms other than the DP mechanism, can act dominantly in high-quality samples with moderately high *T*_g_ (middle 500 °C) as to determine the upper limit of *τ*_s_. We also mention that even the sample with *T*_g_ of 432 °C—having the lowest interface quality among our samples—shows *τ*_s_ of about 4 ns, which is still an order of magnitude longer than that of (100) QWs. This suggests that the Rashba in-plane *B*_eff_ induced by interface imperfections is significantly smaller than the Dresselhaus in-plane *B*_eff_ in (100) QWs. Further investigation is needed for clarifying the mechanisms such as *local* Rashba spin–orbit coupling for limiting *τ*_s_ in an ideal (110) QWs without any crystal imperfections.

## 4. Conclusions

In order to optimize MBE growth conditions to obtain a long photoluminescence lifetime (*τ*_PL_) and electron spin relaxation time (*τ*_s_) in (110)-oriented GaAs/AlGaAs multiple quantum wells (MQWs), we systematically investigated impacts of crystal quality on carrier recombination and spin dynamics in (110) MQWs with growth temperatures in the range of 430 to 600 °C. The MBE growth of (110) MQWs at moderately high growth temperatures (middle 500 °C range) under irradiation of a large number of As atoms provides significant improvements in interface quality, resulting in long *τ*_PL_ (~40 ns) as well as *τ*_s_ (~6 ns) at RT. These findings obtained will be useful information in developing high-performance spintronics devices.

## Data Availability

The data presented in this study are available on request from the corresponding author.
